# Analysis of archaic human haplotypes suggests that 5hmC acts as an epigenetic guide for NCO recombination

**DOI:** 10.1186/s12915-022-01353-9

**Published:** 2022-08-04

**Authors:** Bernett Lee, Samantha Leeanne Cyrill, Wendy Lee, Rossella Melchiotti, Anand Kumar Andiappan, Michael Poidinger, Olaf Rötzschke

**Affiliations:** 1 Singapore Immunology Network (SIgN), Agency of Science Technology and Research (A*STAR), 8A Biomedical Drive, Singapore, 138648 Singapore; 2 Present address: Lee Kong Chian School of Medicine, Nanyang Technological University, 50 Nanyang Avenue, Singapore, 639798 Singapore; 3 Present address: Cold Spring Harbor Laboratory, One Bungtown Road, NY 11724 Cold Spring Harbor, USA; 4 Present address: Murdoch Children’s Research Institute, Royal Children’s Hospital, Flemington Road, Parkville, Victoria 3052 Australia

**Keywords:** Meiotic recombination, Non-crossover recombination, Epigenetic inheritance, Neutral evolution

## Abstract

**Background:**

Non-crossover (NCO) refers to a mechanism of homologous recombination in which short tracks of DNA are copied between homologue chromatids. The allelic changes are typically restricted to one or few SNPs, which potentially allow for the gradual adaptation and maturation of haplotypes. It is assumed to be a stochastic process but the analysis of archaic and modern human haplotypes revealed a striking variability in local NCO recombination rates.

**Methods:**

NCO recombination rates of 1.9 million archaic SNPs shared with *Denisovan* hominids were defined by a linkage study and correlated with functional and genomic annotations as well as ChIP-Seq data from modern humans.

**Results:**

We detected a strong correlation between NCO recombination rates and the function of the respective region: low NCO rates were evident in introns and quiescent intergenic regions but high rates in splice sites, exons, 5′- and 3′-UTRs, as well as CpG islands. Correlations with ChIP-Seq data from ENCODE and other public sources further identified epigenetic modifications that associated directly with these recombination events. A particularly strong association was observed for 5-hydroxymethylcytosine marks (5hmC), which were enriched in virtually all of the functional regions associated with elevated NCO rates, including CpG islands and ‘poised’ bivalent regions.

**Conclusion:**

Our results suggest that 5hmC marks may guide the NCO machinery specifically towards functionally relevant regions and, as an intermediate of oxidative demethylation, may open a pathway for environmental influence by specifically targeting recently opened gene loci.

**Supplementary Information:**

The online version contains supplementary material available at 10.1186/s12915-022-01353-9.

## Background

Only a small fraction of the genome is directly associated with function: nearly 70% is quiescent and function-related regions such as exons, promoter, and enhancer elements are condensed into less than 5% of the total genomic space [1]. The rate and efficiency of genetic evolution would thus arguably be greatly enhanced if molecular mechanisms exist that would direct the induction of genetic variations specifically towards these function-associated regions. On the molecular level, “function” is indicated by the state of the chromatin. The degree of condensation is shaped and maintained by epigenetic patterns, which control the accessibility of loci. It could thus be imagined that elements such as histone and DNA modifications might form a guide to direct the mutation and/or recombination machinery towards active genes and other relevant regions related to gene and chromatin function.

To test this hypothesis we focussed on recombination. Contrary to point mutations, which are mostly caused by random replication errors, it requires an extensive machinery of enzymes and other factors that could be utilized for function-specific targeting. For instance, crossover (CO) recombination is tied to predetermined breakpoints that are primarily defined by the binding motif of the zinc finger protein PRDM9 [2]. However, at least in humans, they appear to avoid functional sites. The deCODE map comprising half a million crossovers identified in 15,000 Icelandic meiosis, indicated significantly reduced recombination rates in exons [3]. Also, transcriptional start sites and most transcription factor (TF) binding sites are largely deprived of CO hotspots [4–6]. A notable exception to this observation is a number of recombination-enriched TF sites in sperm, suggesting that the PRDM9-driven recombination may be targeting certain active or poised promoters in germ cells.

The situation might be different for non-crossover (NCO) recombination. NCO recombination refers to the copying of alleles from one chromatid to the homolog region on the other chromatid, typically covering one or only a few SNPs. It is accomplished by gene-conversion, a process in which short tracks of homologous DNA of about 1kb are copied to fill a gap induced by a double-strand break (DBS) followed by restricted exonuclease digestion [7]. Some additional allelic swaps may derive from multiple rounds of uneven crossover (CO) recombination but these occur only proximal to recombination hotspots [8].

Gene-conversion is an integral part of the DNA damage repair (DDR) mechanism [9]. Stress-induced double-strand breaks (DSBs) are unavoidable and occur in all cell types. In germ cells, they contribute to a large fraction of the inheritable recombination events. While the likelihood of induction of DSBs may depend on factors such as mechanical stress and chromatin structure [10], the recruitment of the DSB repair machinery and the efficiency of the alignment of the homologous regions may be affected by the chromatin environment [11]. As it facilitates the exchange of single alleles, it represents a very effective mechanism for the gradual adaptation of haplotypes [12]. The latter is evident for instance in the concerted evolution of multigene families, a process largely driven by inter-locus gene conversion [13].

Therefore, for this study, we focused on both kinds of NCO recombination events: those occurring in pre-defined recombination hotspots and those that are not locally restricted to a hotspot. We used archaic haplotypes, defined by the sequenced genomes of *Denisovan* hominids [14], to analyze the allelic rearrangements of the human genome during the past 500,000+ years. NCO recombination rates of individual SNPs were then estimated using genotype information of modern humans [15, 16]. The analyses revealed that, contrary to CO recombination, high NCO recombination rates were detected in functionally relevant regions such as exons, 5′- and 3′-UTR, open chromatin, and CpG islands but low rates in introns and intergenic regions. Correlation with ChIP-seq tracks from ENCODE and other published sources indicated a clear association of the rate with open chromatin and a number of epigenetic marks. In particular, the distribution of the DNA modification 5-hydroxymethylcytosine (5hmC) was strongly juxtaposed with the NCO recombination rate, making it a promising mechanistic candidate for a targeted or ‘guided’ form of genetic evolution.

## Results

### Archaic linkage blocks

**Fig. 1:**
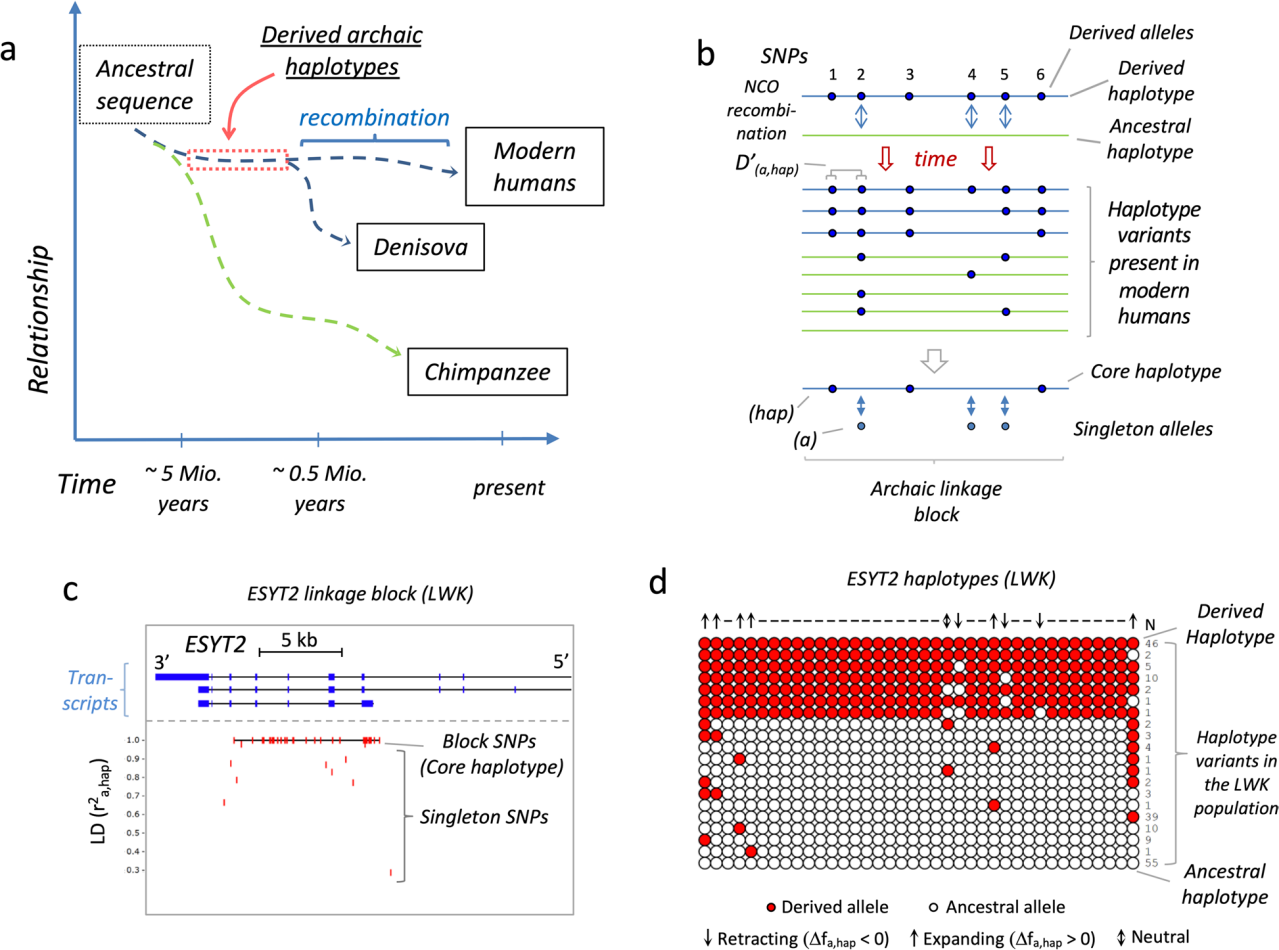
Archaic linkage blocks. **a** Timeline for the formation of archaic haplotypes. As a rough estimate, the chimpanzee line separated from the *h. sapiens* line about 5 million years ago, while the *Denisova h.* split off around 500,000 years ago. This provides a time window of 0.5–5 million years, at which point mutations accumulated in the genome of the common ancestor of *Denisova h.* and *H. sapien*. The derived “archaic” haplotypes formed by these mutations were preserved in *Denisova* and serve as a reference for the recombination events rearranging these haplotypes in the human during a period of at least 500,000 years after separation. **b** Formation of archaic linkage blocks. NCO recombination between the derived archaic haplotype (blue), consisting of the derived alleles of these SNPs (solid circles), and the corresponding ancestral haplotype (green) created a set of haplotype variants that are still present in modern humans. In the given example the archaic linkage block consists of 6 SNPs of which SNPs 1, 3, and 6 are still in perfect linkage (block SNPs). The derived alleles of these SNPs form the core haplotype (hap), which represents the remaining fragment of the derived archaic haplotype. SNPs 2, 4, and 5 are singletons whose alleles had been shifted between haplotypes by NCO recombination. Their association to the derived haplotype is indicated by the linkage disequilibrium (D’_a,hap_) between their derived alleles (**a**) and the core haplotype (hap). **c** Example of an archaic linkage block. The panel shows the location of the SNPs of an archaic linkage block in reference to the intron/exon structure of the ESYT2 gene. In this visualization, the association of the derived alleles to the core haplotype (*r*^2^ = 1) is indicated by the respective *r*^2^_a,hap_ value. The horizontal line connecting the block SNPs indicates the location of the core haplotype; single vertical lines indicate the NCO rate and location of the singleton SNPs. **d** Haplotype composition of the linkage block. The panel displays the haplotype composition of the ESYT2 linkage block defined for the 99 individuals of the LWK cohort. The presence of derived alleles (red circles) and ancestral alleles (white circles) for each haplotype variant is indicated. Numbers indicate the absolute frequency of the haplotype in the cohort; downward and upward arrows on top of the plot respectively indicate retracting and expanding derived alleles, a double arrow indicates a neutral allele that switched the position in several haplotypes without affecting its allele frequency; horizontal lines refer to block SNPs

The linkage analysis was carried out with the genotype data of 99 individuals of the Luhya people from Webuye, Kenya (LWK) [15, 16]. Only those SNPs were considered, for which the derived alleles were found in the genome of the *Denisovan hominid* [14] and the ancestral allele was still present in chimpanzees [17]. This resulted in a dataset of 1.9 million “archaic” SNPs. As an East-African population, the LWK genomes were unaffected by late interbreeding with *Denisova h* [14]. Therefore, most of the derived alleles of these SNPs should have been introduced approximately between 5.0 and 0.5 Mya (i.e., after the separation from chimpanzees but prior to the separation from *Denisova h*.) (Fig. 1a). This provides a time window of more than 500,000 years in which the archaic haplotypes, formed by the derived alleles, could be rearranged by recombination (Fig. 1b). The sequence of these “derived” haplotypes is preserved in the *Denisova* genome, while the alleles of the corresponding ancestral haplotype can be defined by the sequence of allelic states in the chimpanzee genome.

Due to CO recombination, the 1,897,400 archaic SNPs of the LWK cohort were broken down into 237,312 discrete blocks (archaic linkage blocks), each comprising 2 to more than 500 SNPs (Table 1). While the majority of the archaic SNPs in the LWK cohort were found to be still arranged in the original allelic order defined by the *Denisovan* haplotypes (block SNPs), 876,329 SNPs were identified as singleton SNPs, presumably the result of NCO recombination (Fig. 1b). Concordant with the GC bias of gene conversion [8, 22–24], the majority of these singleton SNPs had indeed transmitted G:C over A:T (Additional file 1: Fig. S1a). This applied for 57% of the non-CpG and for 62% of the CpG SNPs, confirming that the allelic rearrangements of singleton SNPs in our database are likely the result of gene conversion events.

**Table 1:** Archaic SNPs in the LWK population (1000 genome project). The table indicates the average number of block SNPs and singletons in the archaic linkage blocks of LWK and summarizes the distribution of the archaic alleles in structurally defined genomic sub-regions as well as in regions defined by ChIP-seq tracks. Numbers indicate the absolute count of archaic SNPs detected in the region; numbers in brackets indicate the percentage in reference to the total amount of the respective SNP type

Type of archaic SNP	Average number of SNPs per linkage block*	Distribution of archaic SNPs								
		Complete genome	Genic region	HLA genes (MHC I and II)	CpG Island (CGI)	Open chromatin (DNase I)^a^	Recomb. hotspot (DCM1)^b^	H3K4me3/H3K27me3 (bivalent)^a^	5mC tracks^c^	5hmC tracks^d^
Block SNP	4.30	1021071	379447	5278	9105	178314	10424	33888	776586	11914
		(53.81)	(37.16)	(0.52)	(0.89)	(17.46)	(1.02)	(3.32)	(88.60)	(1.17)
Singleton	3.69	876329	353376	3025	15175	196934	56698	49900	656974	22400
		(46.19)	(40.32)	(0.35)	(1.73)	(22.47)	(6.47)	(5.69)	(86.90)	(2.56)
Total	8.00	1897400	732823	8303	24280	375248	67122	83788	1433560	34314
		(100.00)	(38.62)	(0.44)	(1.28)	(19.78)	(3.54)	(4.42)	(87.81)	(1.81)

*237,312 linkage blocks (2–500 SNPs, average size: 20.3 kb)

^a^ENCODE-tracks [18, 19]

^b^Human testis [20]

^c^H1 stem cells, partial coverage [1]

^d^H1 stem cells [21]

One example of an archaic linkage block is shown in Fig. 1c. The large block consists of 28 block SNPs and 10 singleton SNPs. They are located close to the 3′ end of ESYT2 and cover about 10kb, including 5 exons, of the gene. The haplotype variants formed by these SNPs are shown in Fig. 1d. Intact versions of archaic and ancestral haplotypes are generally the most common haplotype variants found in archaic linkage blocks (Additional file 1: Fig. S1b). In the case of the ESYT2 block, 46 of the haplotypes in the LWK cohort are identical to *Denisovans,* while 55 matched chimpanzees, representing perfectly preserved derived and ancestral haplotypes, respectively. Each of the remaining 97 haplotypes had allelic swaps in up to 3 SNP positions due to NCO recombinations.

The NCO recombination rates of these singleton SNPs were estimated by determining the linkage disequilibrium (LD) of their derived allele (a) in reference to the respective core haplotype (hap) (Fig. 1b). As *r*^2^ is biased by the allele frequencies, for this study D' has been selected as a measure for the LD. In the following, 1 – D’^2^_a,hap_ was therefore used to indicate the “NCO rate” of these singleton SNPs, a proxy of their recombination rate.

### Chromatin and genic sub-regions

**Fig. 2:**
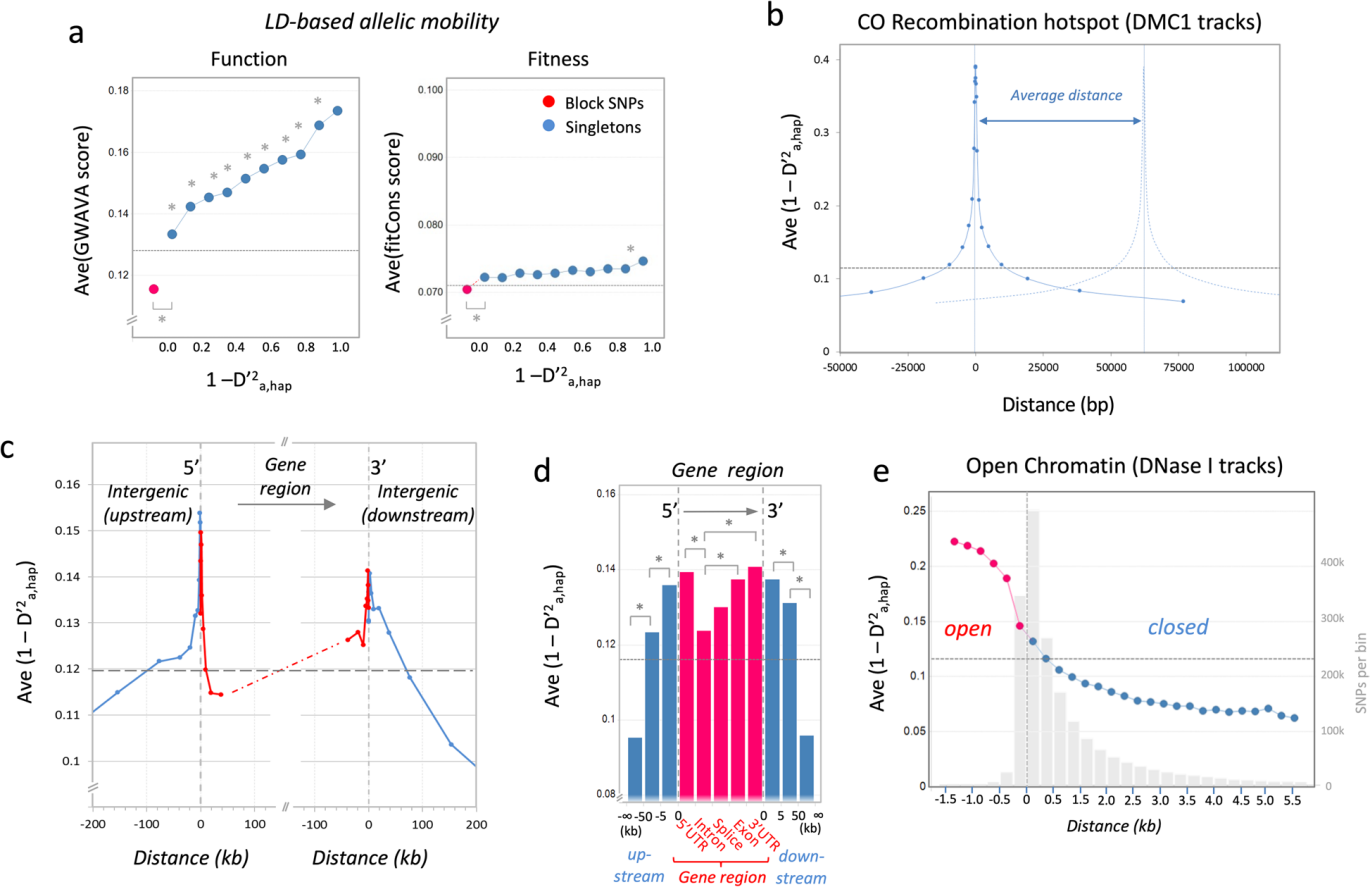
Correlation of the NCO recombination rate with functional genomic parameters. **a** Function vs. fitness. The NCO rate 1 – D’^2^_a,hap_ was used as a proxy for the NCO recombination rate. The correlation of this parameter of about 1.9 million archaic SNPs is shown for function-associated GWAVA scores (left panel) and fitness-associated fitCons scores. The plot displays the average scores for about 1.0 million block SNPs (red dot) and 900,000 singleton SNPs (blue dots). The singleton SNPs were binned according to the 1 – D’2_a,hap_ value defined for the LWK population. Dashed horizontal lines indicate the average scores of the entire set of archaic SNPs. Asterisks indicate significant differences between data point pairs (*p* < 0.05; Mann-Whitney U test). **b** Meiotic CO recombination hotspots. The solid line represents the average NCO rate (1 – D’2_a,hap_) plotted in reference to the distance of the alleles to meiotic CO recombination hotspots (defined by DMC1 ChIP-Seq tracks of human testis [20]). The dashed line of the second peak represents the hypothetical NCO rate in reference to a second recombination hotspot located at the average distance of about 67 kb. **c** Gene boundaries. The average NCO rate (1 – D’2_a,hap_), is plotted in reference their distance to the boundary of the closest annotated gene. Peaks in NCO rate are evident at both 5′- and 3′-boundaries (indicated by dashed vertical lines). Gene regions are marked in red, intergenic regions in blue; dashed horizontal line represents the average NCO rate of the entire set of archaic alleles. (**d**) Genic sub-regions. Gene regions (red) were further delineated into their sub-regions (5′UTRs, introns, splice sites, exons and 3′UTRs); intergenic regions (blue) were divided according to their distance to the boundaries both upstream and downstream of the genic region (0–5 kb, 5–50 kb, >50kb). Bars represent the average NCO rate (1 – D’^2^_a,hap_) in the respective region. Asterisks indicate significant differences between the indicated bars (*p* < 0.05; Mann-Whitney *U* test). **e** Open chromatin. The line chart depicts the association of the NCO recombination rate with open chromatin. The average NCO rate (1 – D’^2^_a,hap_) is plotted in reference to the distance of the alleles to the boundary of open chromatin (defined by ENCODE tracks of DNase I sensitivity). Gray bars indicate the number of archaic SNPs in the respective distance-bin

In an initial attempt to identify links between NCO recombination rate and “function,” we correlated the NCO rate (1 – D’^2^_a,hap_) of our archaic singleton SNPs with “Genome-wide annotation of variants”-scores (GWAVA) [25]. GWAVA is a tool which aims to predict the functional impact of non-coding genetic variants. It is based on a wide range of annotations of non-coding elements (largely from ENCODE/GENCODE), along with genome-wide properties such as evolutionary conservation and GC content. As the genetic variation is also likely to be affected by selection, in parallel, we correlated the data set with “Fitness consequence of functional annotation scores” (fitCons) [26], which are indicative of the putative fitness contribution of a SNP. Although direct correlations with the entire data set were weak (rho values of the Spearman’s rank correlation were only 0.07 for GWAVA and 0.01 for fitCons scores), a correlation with the average scores of NCO rate bins revealed a significant trend for GWAVA-scores and no association with fitCons scores (Fig. 2a). The failure to detect any major impact by natural selection was not due to compromised databases, as a strong, locus-specific, association with fitCon scores was detected, when the allele set was restricted to SNPs located in the MHC genes (Additional file 1: Fig. S2).

To further characterize the functional link between NCO recombination and “function,” we correlated the NCO rate parameter 1 – D’^2^_a,hap_ with annotated regions in the genome (Table 1). A particularly strong influence on the NCO recombination rate was also expected to be observed for meiotic recombination hotspots. While these hotspots primarily represent pre-determined breakpoints for CO recombination, they often also serve as initiation sites of NCO recombination [8, 27, 28]. DMC1 is a homolog of the bacterial strand exchange protein RecA and acts as a specific marker of these hotspots [29, 30]. The NCO rate correlation with DMC1 loci in human testis defined by ChIP-Seq [20] indeed revealed extremely high 1 – D’^2^_a,hap_ values for alleles located proximal to the binding sites of DMC1 (Fig. 2b). The effect, however, was very local: at a distance of approximately 1000 bp from the DMC1 binding sites, the NCO rate declined to half. With an average spacing of about 65kb, the influence of hotspots is, therefore, restricted to less than 5% of the SNPs (Table 1). Due to the erosion of PRDM9 motifs over time [31] and the incomplete coverage of the various PRDM9 alleles [32], the detected number of SNPs affected by these hotspots is likely to be an underestimate.

CO hotspots are mostly located in regions that direct genetic recombination away from functional genomic elements [33] with reduced rates observed in exons [3, 6]. Contrary to this notion, for NCO derived singleton SNPs a correlation with the distance to the closest gene revealed a particularly sharp peak of NCO rate proximal to the 5′ boundary and, to a weaker extent, also at the 3′ boundary of annotated gene regions (Fig. 2c). Further delineation of genic sub-regions corroborated this finding with high NCO rate values observed in the 5′- and 3′-UTRs as well as in the respective flanking regions located upstream and downstream of the genes (Fig. 2d). We also observed a particularly high NCO rate in exons, followed by splice sites and introns (Fig. 2d). In intergenic regions, the NCO rate dropped with increasing distance to the gene boundaries. A correlation with ENCODE tracks of DNase I sensitivity [18, 19] further revealed an increase in NCO rate in regions of open chromatin (Fig. 2e). This applies particularly for alleles in longer open stretches where a sharp NCO rate increase was observed for alleles located more than 500bp from the boundaries. However, this set comprised of less than 0.5% of the total SNPs. Furthermore, in line with prior reports [34], the recombination rate was also found to be directly associated with the local GC content and CpG islands (Additional file 1: Fig. S3a, b).

### Epigenetic marks

To determine if the NCO recombination rate is influenced by epigenetic histone modifications, we superimposed the NCO rate (1 – D’^2^_a,hap_) with encode ChIP-Seq tracks of various histone modifications (H3K27me3, H3K4me1, H3K4me3, H3K4me2, H3K9ac, H3K27ac, H3K79me2, H3K36me3, H3K9me3). In order to assess their impact independently for each genomic location, separate NCO rate correlations were carried out for seven genic sub-regions (intergenic, < 5kb upstream, 5′ UTR, intronic, exonic, 3′-UTR, and > 5kb downstream regions).

**Fig. 3:**
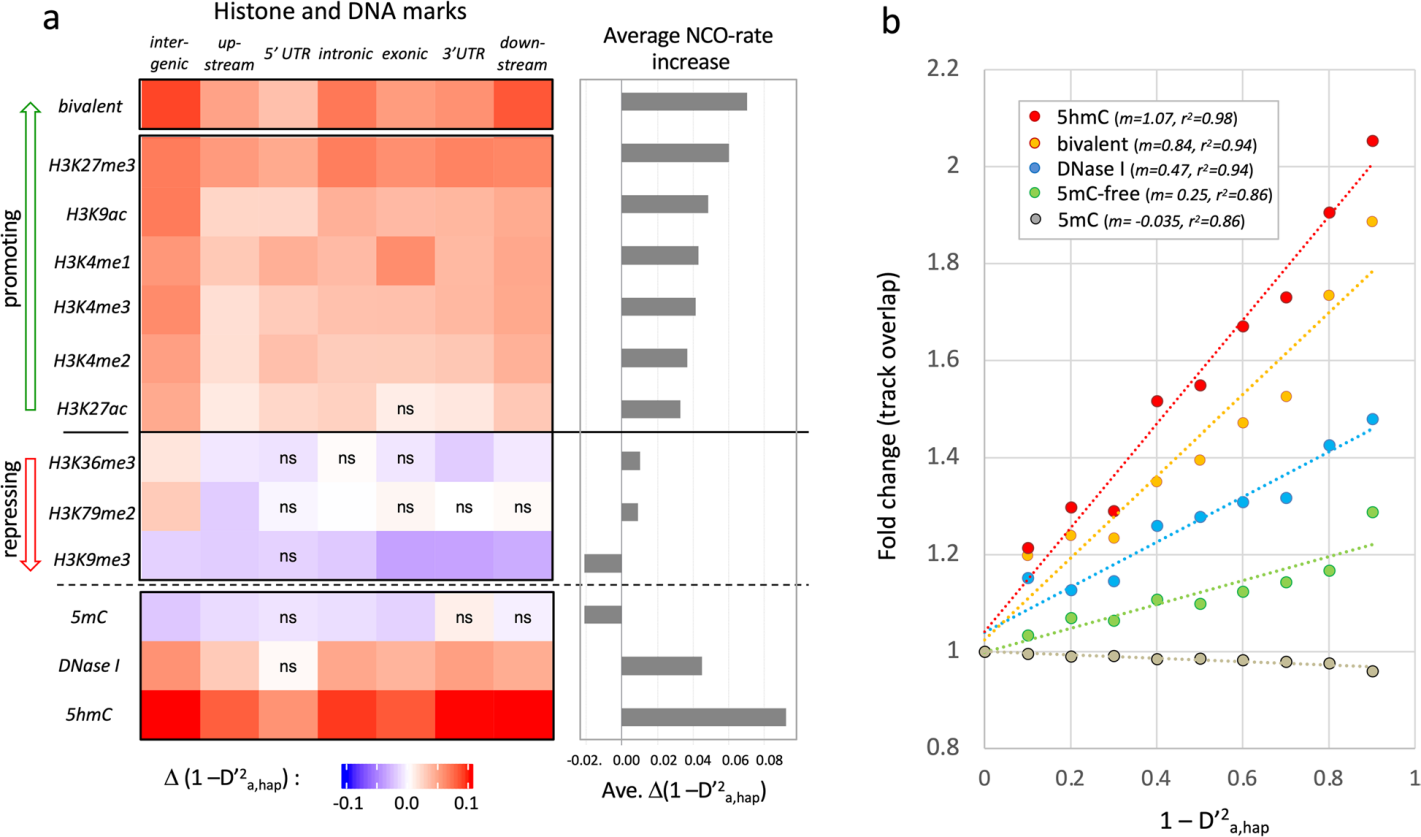
Correlation of the NCO recombination rate with epigenetic marks. **a** Heatmap and cumulative scores of histone and DNA marks. The impact of epigenetic marks on the NCO recombination rate was assessed by determining the average NCO rate (1 – D’^2^_a,hap_) for alleles located on tracks of histone marks (bivalent H3K27me3 & H3K4me1, H3K27me3, H3K4me1, H3K4me3, H3K4me2, H3K9ac, H3K27ac, H3K79me2, H3K36me3, H3K9me3), of 5-methylcytosine (5mC), and 5-hydroxymethylcytosine (5hmC), as well as of open chromatin defined by DNase I sensitivity. To avoid any bias due to the genomic location of the SNPs, the data set was divided into the respective genic sub-region (intergenic, upstream > 5 kb, 5′ UTR, intronic, exonic, 3′ UTR, downstream < 5 kb). Each combination of epigenetic mark and genic sub-region were tested independently using Mann-Whitney *U* test to determine if the overlap with the respective mark led to a significant difference in the NCO rate (Additional file 3: Table S2). Left panel: The color code in the heat map indicates the deviation from the average the NCO rate Δ(1 - D’^2^_a,hap_) of the respective sub-region in reference to regions without any mark (ranging from − 0.1 (blue) to 0.1 (red)). “ns” indicates a non-significant association; solid horizontal line separates recombination promoting marks (green arrow) from recombination repressing marks (red arrow). Right panel: The bars represent the average increase in the NCO rate score for each of the marks across the entire genome. ChIP-Seq tracks of histone marks were compiled from ENCODE data of various tissues and cell lines while tracks of 5mC, DNase I, and 5hmC marks were derived from the embryonic stem cell H1 [1, 21]. CO recombination hotspots marked by DCM1 tracks were excluded from the analysis. **b** Fold change in track overlap. The fold change in the overlap with 5hmC tracks (red), H3K27me3/H3K4me3-defined bivalent regions (orange), open chromatin-related DNase I tracks (blue) and 5mC-free tracks (green) and 5mC-containing tracks (gray) in response to the increase in the average NCO rate (1 – D’^2^_a,hap_) is shown. The dots indicate the fold change for the binned NCO rate average. Only singleton SNPs are shown. Dashed lines represent linear regressions; slope coefficient (m) and r^2^ value are indicated. Fold change for bivalency and DNase I was compiled from the ENCODE data set while 5mC and 5hmC were compiled from data provided for the H1 stem cell

Notably, all of the analyzed histone marks showed association with the NCO rate in at least some of the genic subregions (Fig. 3a, Additional file 3: Table S2). Direction and extent, however, varied strongly with the type of methylation. Depending on the general direction, the impact of the modification could be classified either as “recombination-promoting” (H3K27me3 >> H3K4me1, H3K9ac, > H3K4me3, H3K4me2 > H3K27ac) or “recombination-repressing” (H3K9me >> H3K36me3, H3K79me2). For individual histone marks, the strongest positive association was observed for H3K27me3, while the strongest negative association was observed for H3K9me3. The highest NCO rate values were detected in bivalent regions—tagged by both H3K27me3 and H3K4me3 marks (Fig. 3a, Additional file 1: Fig. S3c). Bivalent histone modifications are hypothesized to “poise” silenced genes for rapid activation [35], a state, which, according to this data, seems to prime the loci for NCO recombination.

To analyze also epigenetic DNA modifications, ChIP-Seq tracks for 5-methyl-cytosine (5mC) and 5-hydroxymethyl-cytosine (5hmC) were downloaded together with matching DNase I-sensitivity tracks. In contrast to the histone tracks, which were compiled from a number of different cell lines, the DNA tracks were derived from for the embryonic stem cell H1 [1, 21]. In line with expectation, an increased NCO rate was observed in open DNase I-assessable chromatin, while reduced recombination rates were detected in regions enriched for 5mC, indicating the closed state of chromatin (Fig. 3a). The strongest association however was observed for 5hmC, an intermediate of oxidative C-demethylation and marker of recently opened chromatin (Fig. 3a). While DNase I showed a positive association in nearly all genic sub-regions (Fig. 3a, left panel), the average score was substantially weaker than the score of H3K4me1, H3K27me1, and of bivalent regions (Fig. 3a, right panel), suggesting that “openness” alone may not be sufficient to promote recombination.

In all analyzed sub-regions, the gain in NCO rate was the highest when the alleles overlapped with 5hmC tracks (Fig. 3a). The average score of 5hmC in fact eclipsed the scores of all other tested markers, suggesting that 5hmC was the marker most closely associated with the local NCO rate (Fig. 3a, right panel). Plots of the NCO rate vs. the fold change of the track overlap revealed a nearly linear correlation for all markers. The coefficient was 1.09 for 5hmC and 0.85 for bivalency, compared to only 0.4 and 0.24 for DNase I and 5mC, respectively, confirming the strong impact of 5hmC and bivalency on the recombination rate (Fig. 3b).

### NCO rate and 5hmC content in epigenetic sub-regions

**Fig. 4:**
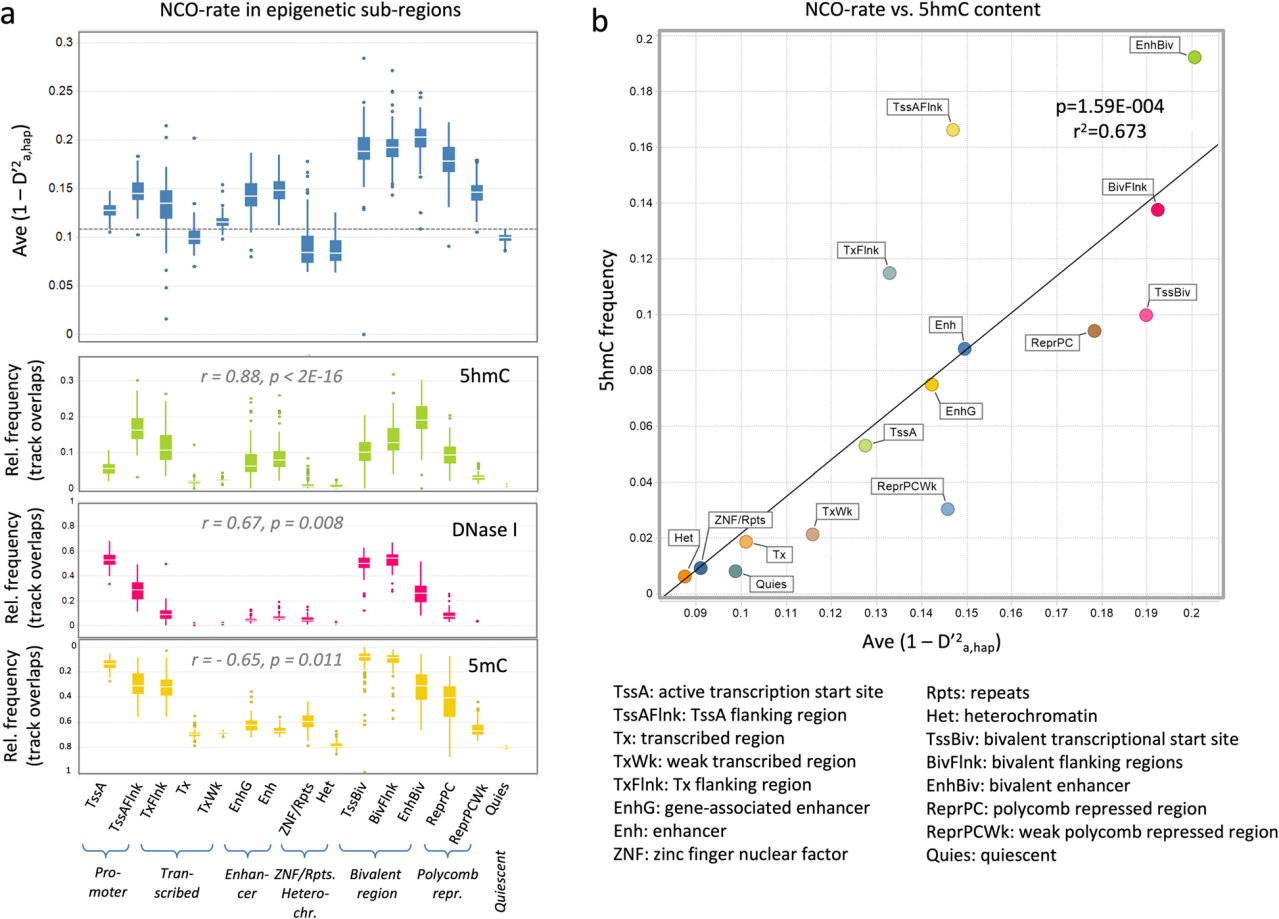
Association of 5hmC and NCO recombination rates. **a** Association in epigenetically defined regions. NCO rate, chromatin state and DNA marks are shown for 15 epigenetic sub-regions comprising active transcription start sites (TssA), TssA flanking regions (TssAFlnk), transcribed regions (Tx), weak transcribed regions (TxWk), Tx flanking regions (TxFlnk), gene-associated enhancer (EnhG), enhancer (Enh), zinc finger nuclear factor regions (ZNF), repeats (Rpts), heterochromatin (Het), bivalent transcriptional start site (TssBiv), bivalent flanking regions (BivFlnk), bivalent enhancer (EnhBiv), polycomb repressed region (ReprPC), weak polycomb repressed region (ReprPCWk) and quiescent regions (Quies) [1]. Histone marks are shown in Additional file 1: Fig. S4. The calculations were carried out separately for each of 111 reference genomes using the entire data set of 1.9 million archaic SNPs. The boxplots indicate for each region the average NCO rate (1 − D’^2^_a,hap_), as well as the average track overlap of these SNPs with 5hmC marks, DNase I-defined, and 5mC-defined open chromatin. The NCO rate was calculated using the data from the LWK cohort, while 5mC, DNase I, and 5mC tracks were defined for the H1 stem cell line [1, 21]. The r- and p-values refer to a Spearman rank correlation carried out between these relative frequencies of the marks and the respective NCO rate data. **b** Correlation of NCO recombination rate with the 5hmC frequency. The plot shows the average NCO rate and corresponding track overlap of the SNP with 5hmC marks for each of the 15 epigenetically defined subregions. The line was derived by Pearson correlation, the *p* and *r*^2^ values are indicated

To confirm the impact of the epigenetic environment on the local NCO rate we used an independent data set from the NIH Roadmap Epigenomics Consortium that comprises the epigenomes of 111 tissues, cells, and cell lines [1]. It allows annotation of 15 epigenetically defined sub-regions defined mostly by the presence of characteristic histone marks (Additional file 1: Fig. S4). Based on this annotation, NCO recombination rates (as defined by NCO rate) could be determined independently for each of these sub-regions. These rates could then be correlated with the fraction of open and closed chromatin (defined by DNase I and 5mC, respectively) and the overlap with 5hmC tracks (Fig. 4a). Strikingly similar patterns emerged when comparing NCO rate in the various epigenetic regions (Fig. 4a, upper panel) with the relative track-overlaps of 5hmc (middle panel). The Spearman correlation between the 5hmC distribution and NCO rate revealed *r* = 0.88 and *p* = 2E-16. In line with the prior data the association with DNase I-defined open chromatin was weaker (*r* = 0.67, *p* = 0.008). The same applied also for the 5mC tracks, which inversely mirrored the DNAse I pattern (*r* = − 0.65, *p* = 0.011).

The direct correlation of the between NCO rate of the subregions and the respective 5hmC frequency revealed an *r*^2^ value of 0.67 (Fig. 4b). Within these subregions, the highest NCO rate and 5hmC frequency values were detected in bivalent regions marked by the presence of both H3k27me3 and H3K4me3. This applied for “poised” versions of promoter (TssBiv), enhancer (EnhBiv), and flanking regions (BivFlank), while the “non-poised” active counterparts TssA, EnhG, Enh, and TssAFlnk were ranking substantially lower. Slightly lower values were also detected in Polycomb-repressed regions (ReprPC), while the lowest values were detected in ZNF/Repeat and Heterochromatin regions. Low values were also detected for alleles in transcribed regions (Tx, TxWk, TxFlnk). This however may be due to the fact that these regions largely comprise of introns, which are associated with low NCO rate (compare Fig. 2d).

A more detailed analysis on the frequency of 5hmC marks revealed that they were indeed strongly enriched in all regions identified before to be associated with increased recombination rates. This applies for bivalent regions, open chromatin, CpG islands (CGI), and DMC1 tracks (Additional file 1: Fig. S5a). Plots representing the association with the GC content further revealed similar maxima curves for both 5hmc enrichment and NCO rate (Additional file 1: Fig. S5b, left panel) and also a peak in 5hmC depositions in CpG islands closely matched the sharp NCO rate increase observed at these sites (Additional file 1: Fig. S5b, right panel). Analysis of the 5hmC density in genic regions revealed that it spiked at the 5′- and, to a lesser extent, the 3′-boundaries of genes (Additional file 1: Fig. S5c, left panel), closely mirroring the NCO rate profile shown in Fig. 2c. Also, the breakdown of genic sub-regions revealed a similar pattern as described for NCO rate: the 5hmC density was highest at 5′-UTR and gradually decreased in intergenic regions with increasing distance to the gene boundaries. It was low in introns, increased in splice sites, and was highest in exons (Additional file 1: Fig. S5c, right panel). Thus, 5hmC seems to be prominently present in all the functionally relevant loci that, according to our analyses, are associated with an increased NCO recombination rate.

## Discussion

The correlation between epigenetic modifications from modern day human cells, and NCO rate in this analysis, certainly is an extrapolation. However, by analyzing NCO events in archaic human haplotypes, we established that the local recombination rates were aligned with the putative functional value of the respective genomic region. Elevated rates were observed in the 5′ and 3′ UTRs, as well as in splice sites and exons, while they decrease in intergenic regions with increasing distance to the gene boundaries. Increased rates were also found in open chromatin, regions with high GC-contents and CpG islands, and especially in bivalently “poised” regulatory regions. While various histone marks associated with the rate in either a positive or negative way, the closest correlation was observed for the DNA mark 5hmC. It was enriched in all functionally relevant regions associated with an increased NCO recombination rate.

Variations in the local recombination rate have been described before, although none of these reports provided a direct link to “function.” For instance, it is well established that the recombination rate in telomeric regions is elevated in males compared to females [36], a phenomenon known as heterochiasmy [37]. A comprehensive study of the deCODE consortium of 15,257 Icelandic parent–offspring pairs also indicated that, contrary to our analyses, human recombination appeared to be repressed within gene regions [3]. Their analysis, however, covered mostly alleles that had been swapped by chromosomal crossover (CO). For CO, the genomic location of double-strand breaks (DSB) is primarily defined by the binding motif of the zinc finger domains of PRDM9 [2, 38, 39]. Notably, in dogs where the orthologue of PRDM9 is non-functional [40] the recombination pattern is much more consistent with our results: elevated rates in promotor regions close to the transcriptional start site and around CpG islands [41]. Likewise, in mice lacking functional PRDM9, the DSB-hotspots are enriched in promoter- and CpG-rich regions [33]. By restricting our analysis to the NCO recombination of singleton SNPs, we eliminated the bulk of PRDM9-based CO-recombination events. Similar to the recombination patterns in PRDM9-deficient animals, the allelic rearrangement now preferably targets functional elements.

Similar to NCO also CO hotspots appear to be enriched in bivalent regions [42]. In the case of CO recombination this is directly linked to PRDM9, as it acts as germ line-specific H3K4 methyltransferase [2, 39, 43, 44]. For NCO recombination positive associations were observed for H3K27me3 as well as a number of other H3 modifications, such as H3K4me1 and H3K9ac, while negative associations were detected for H3K36me3 and H3K9me3. Similar to CO, a particularly strong correlation was observed for bivalent regions marked by both H3K4me3 and H3K27me3. The analysis of epigenetic sub-regions further revealed increased NCO recombination rates in the ‘poised’ versions of enhancer, promoter and flanking regions. Notably, a prior study indicated that in germ-line cells, poised genes often act as ancient regulators [45]. As the lineage-specific poising correlates with evolutionary innovations, it is intriguing that both NCO and CO recombination seems to be directed to bivalent regions.

In contrast to histone marks that associated in a composite manner, open chromatin and, even more profoundly, 5hmC correlated directly with the NCO recombination rate. 5hmC is a unique epigenetic mark [46] influencing chromatin structure and DNA Binding Protein (DBP) interactions [47, 48]. It is strongly associated with bivalent chromatin [49] as well as with promoters bearing the polycomb repressive mark, H3K27me3 [50–54]. 5hmC is also enriched in genic regions [55, 56] with increased levels in exons [21, 56], transcription factor binding sites [21, 55], and 5′UTRs and TSSs of genes [49]. Based on our study, the local density of the mark closely corresponds to the frequency of NCO recombination events: both parameters are increased in bivalent regions, open chromatin, CpG islands, and recombination hotspots, they peak in exons and UTRs and follow a very similar trend in epigenetic sub-regions (bivalent >> polycomb-repressed >> active enhancer > promoter >> transcribed regions > quiescent > ZNF/repeats, heterochromatin).

**Fig. 5:**
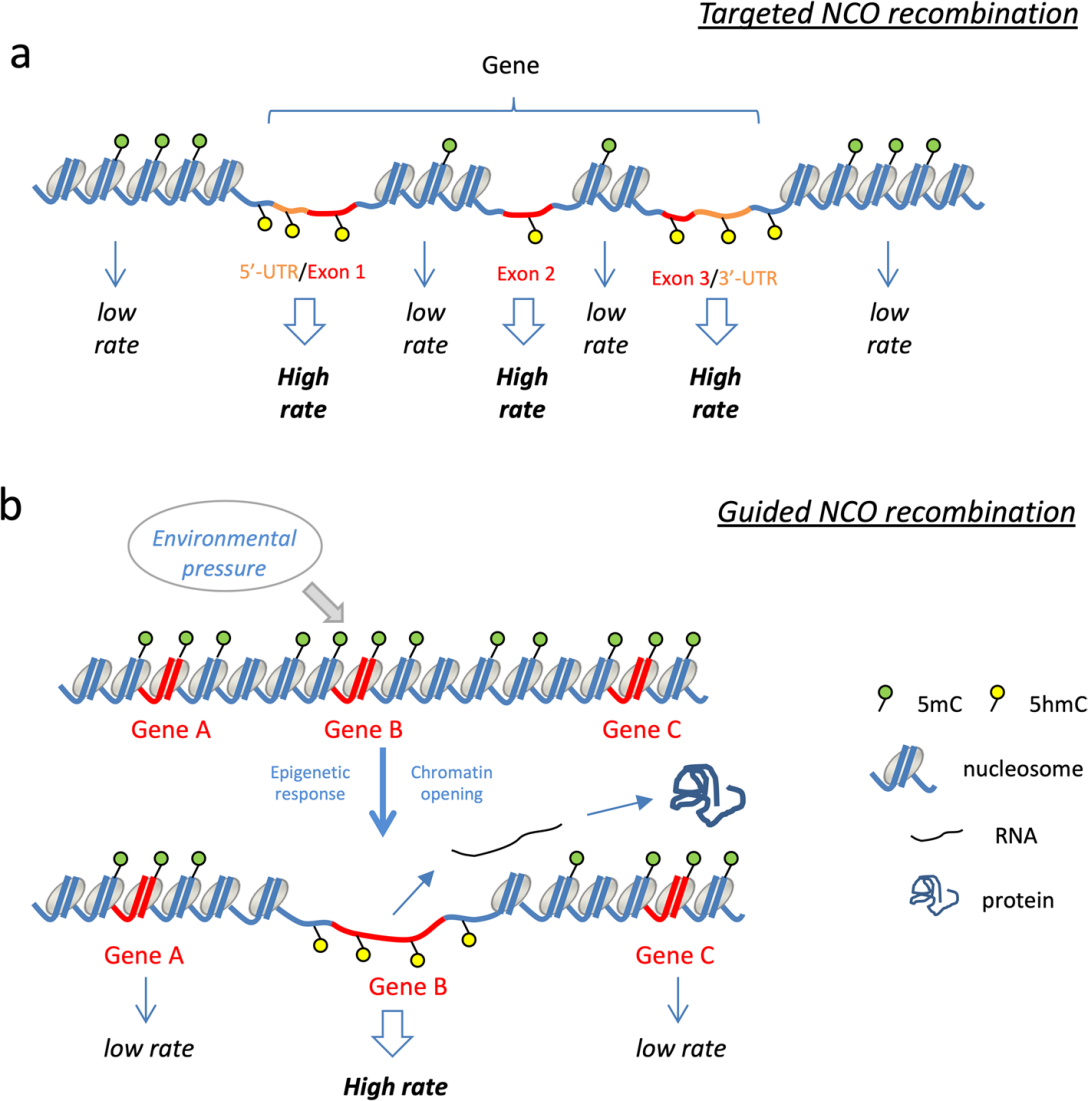
Model of targeted and guided NCO recombination. **a** Targeted NCO recombination. 5hmC is enriched in functional regions such as UTRs and exons (compare Fig. 4b). Thus, utilization of marks like 5hmC would allow to target the NCO recombination machinery towards function-related loci, which would in turn undergo more frequent recombination than quiescent or non-functional regions. **b** Guided NCO recombination. 5hmC is also a product of oxidative C-demethylation and indicative of recently opened chromatin. As this process can also occur in response to environmental signals, newly formed 5hmC marks would direct the recombination machinery preferably to these sites. In this model, the environmental pressure would therefore “guide” genetic evolution in a Lamarckian sense

One plausible mechanism linking the 5hmC mark to NCO recombination could be that it acts as a tag for induced doublestrand breaks. A candidate to execute this cut is ENDOG, a ubiquitous eukaryotic restriction enzyme that reportedly creates substrates for genetic recombination by cleaving 5hmC-modified DNA [57]. Studies with ENDOG −/− mice have established a role for this enzyme in the somatic recombination required for immunoglobulin class switching [58] and in somatic cells, it co-localizes with 53BP1 and γH2AX, two key components of the DNA damage repair (DDR) complex [59]. As the DDR pathway is also active in the germ line, 5hmC-directed gene conversion may therefore act as a recombination pathway parallel to the canonical CO recombination pathway, targeting only the predetermined recombination sites. Due to the enrichment of the mark in exons, splice sites, UTR, enhancer, or CpG islands, it would direct NCO recombination towards these function-related regions (Fig. 5a).

Lastly, as a product of the cytosine-demethylation pathway [60], 5hmC is also a dynamic marker of recently opened loci [61]. Oxidative demethylation can occur in response to environmental stress, as observed, for instance, in dendritic cells exposed to *M. tuberculosis* infection [61, 62]. Provided, that some of these signals are transmitted also into the germ line cells, the epigenetic response could direct the NCO recombination machinery towards the respective stress-response genes, facilitating a “guided” evolution in the Lamarckian sense (Fig. 5b). It is already established that “transgenerational epigenetic inheritance” (TEI) allows the transfer of environmentally induced phenotypes through the germ-line [63–65]. While the epigenetic imprintment is lost after a few generations, a link between genetic recombination and TEI may allow to convert “soft” epigenetic marks into “hard” genetic variation [66]. Further experimental studies need to be undertaken to prove the validity of this intriguing hypothesis.

## Conclusion

In this study, we conducted a linkage analysis of human SNPs and calculated the rates of NCO recombination. Using publicly sourced epigenetic data, we found that certain epigenetic parameters and, in particular, 5hmC deposition marked regions of heightened NCO rate. 5hmC being a dynamic mark, susceptible to environmental influence, could serve as a gateway for environmental impact on evolution. Finally, we propose 5hmC distribution may provide an epigenetic model of targeted recombination and guided evolution.

## Methods

### Definition of archaic SNPs

Archaic SNPs are defined here as the set of human SNPs where the derived and ancestral allele is present respectively in the sequence of *Denisova hominins* [14] and chimpanzees [17]. To minimize errors due to ancient DNA damage only those SNPs were considered that were found to be homozygous in *Denisova*. Human alleles shared with *Denisova* were obtained as VCF files (http://cdna.eva.mpg.de/Denisova/VCF/hg19_1000g/ [14]), of which all variants annotated in the 1000 Genome Project were extracted [15, 16]. For each SNP, the ancestral allele is defined to be the one found in the chimpanzee genome [17] and the derived ‘archaic’ allele being the one found in the *Denisovan* genome. This resulted in a total of 1,897,400 archaic SNPs. All positional information is based on human genome hg19. The complete dataset used in this study has been deposited as a tab-delimited text file together with an Excel metadata file describing all the fields within the tab-delimited text file at 10.7910/DVN/WA7IUK.

### Definition of archaic linkage blocks

The linkage analysis was carried out with phase 3 phased genotype data for 99 individuals of the LWK population (Luhya in Webuye, Kenya) provided by the 1000 genome project [15, 16]. The linkage disequilibrium was computed using a Java program, which allowed for computation based on the actual frequency of the derived allele (*f*_*a*_) instead of just the minor allele frequency (maf). *r*^2^ values were determined for all possible pairs of derived alleles (*r*^*2*^_*a1,a2*_), located within a window of 200,000 bp, using the following equations (with P_a1,a2_ representing the probability of observing the derived alleles a1 and a2 on the same DNA strand):

$${D}_{a1,a2}={P}_{a1,a2}-\left({f}_{a1}\times {f}_{a2}\right)$$

$${r}_{a1,a2}=\frac{D_{a1,a2}}{\sqrt{f_{a1}\times \left(1-{f}_{a1}\right)\times {f}_{a2}\times \left(1-{f}_{a2}\right)}}$$

This matrix allowed for the identification of the sets of perfectly linked SNPs (block-SNPs). Their derived alleles represent the preserved core-haplotype of the original archaic haplotype found in *Denisovan*. The linkage analysis of derived alleles was carried out in reference to the core-haplotype (*r*^2^_a,hap_). Archaic linkage blocks were then formed by assigning each of the remaining singleton SNPs of the archaic SNP set (linked SNPs), to the core haplotype with the closest linkage (i.e., highest *r*^2^_a,hap_ value). This resulted in 237,313 core-haplotypes comprising of 1,021,071 SNPs with 876,193 singleton SNPs in linkage (136 singleton SNPs were omitted as there were no core-haplotypes within 200,000bp)

### Definition of allele frequencies and NCO rate

Allele frequencies of archaic SNPs of the studied LWK population are expressed as an absolute frequency of the derived allele (*f*_*a*_) as well as a normalized frequency (*Δf*_*a,hap*_) defined by the difference between the frequency of the derived (*f*_*a*_) and the associated core-haplotype (*f*_*hap*_). The latter was also used as indicator of expansion or retraction of the derived allele, which was respectively indicated by a positive or negative *Δf*_*a,hap*_ value. For accurate phasing, *Δf*_*a,hap*_ was defined by *f*_*a*_ *– f*_*hap*_ in case of a positive value for r_a,hap,_ while in case of a negative value *Δf*_*a,hap*_ was defined by (1 - *f*_*a*_ ) *– f*_*hap*_. The LD-based NCO rate, serving as a proxy for the NCO recombination rate, was defined as 1 – (D’_a,hap_)^2^ (indicated in the text as 1 – D’^2^_a,hap_). D’_a,hap_ was computed using the following formula:

$$D_{max}=\left\{\begin{matrix}max \begin{Bmatrix}-(f_{a1} \times f_{a2}), -(1-f_{a1}) \times (1-f_{a2})\end{Bmatrix} & when\ D_{a1,a2} < 0\\ min \begin{Bmatrix}f_{a1} \times (1-f_{a2}), (1-f_{a1}) \times f_{a2}\end{Bmatrix} & when\ D_{a1,a2} > 0\end{matrix}\right.$$

$$D'_{a,hap} = \frac{D_{a1,a2}}{D_{max}}$$

### Assessment of the GC bias

For the analysis of the GC bias only those allele pairs were selected that were characterized by a linkage disequilibrium of D’^2^_a,hap_ = 1 and *r*^2^_a,hap_ <1. In this case, only 3 allelic combinations existed in the studied population, thereby representing the haplotype sets formed by a single NCO recombination event. Δf_a,hap_ > 0 indicated here the expansion of the derived allele into the ancestral haplotype, while Δf_a,hap_ < 0 indicated the replacement of a derived allele by the corresponding ancestral allele:

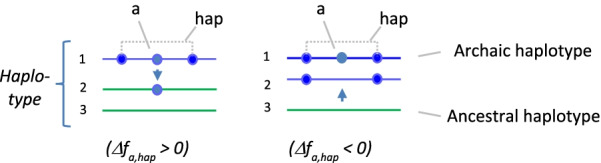



To minimize the influence of genetic drift, only SNPs with a minor allele frequency > 0.2 were considered. Of these, a total of 370,000 non-CpG SNPs and 230,000 CpG SNPs represented heterozygous G:C/A:T pairs. Counting of the SNPs, in which the G:C or the A:T allele was transmitted, revealed respectively 210,000 and 160,000 non-CpG SNPs and 140,000 and 90,000 CpG SNPs, translating into a GC bias of 0.57 (non-CpG SNP) and 0.62 (CpG SNP).

### Frequency of intact ancestral and archaic haplotypes in modern humans

Complete haplotype sets of the 237,312 archaic linkage blocks were generated by downloading the sequence information of the 198 haplotypes of the LWK cohort from the 1000 genome project. The haplotypes, generated by statistical phasing, comprised only ancestral and derived alleles of archaic SNPs. For each block, the relative frequency of perfectly preserved archaic haplotypes (entirely consisting of derived alleles) and of perfectly preserved ancestral haplotypes (entirely consisting of ancestral alleles) was determined. The same approach was also used to determine the relative haplotype frequency of mixed haplotypes binned according to the relative fraction of derived alleles (0 < × < 1, increment: 0.1).

### Correlative parameters

Each archaic SNP present in the LWK data set was annotated with a comprehensive set of parameters (details about the data sources are found in Table S1). Frequency of the derived allele (f_a_), normalized allele frequency (Δf_a,hap_), and LD-based NCO rate (expressed as 1 – D’^2^_a,hap_) were calculated as described above using the genotype data of the east African LWK cohort (99 individuals) [15, 16]. Function-related GWAVA (“Genome-wide annotation of variants”) scores [25] were downloaded together with fitness-related fitCons (“fitness consequences of functional annotation”) scores [26] from the respective sites and assigned to each of the analyzed SNPs. Gene annotation, as well as location of genic sub-regions was determined for each SNP using snpEff (PubMed ID 22728672). The average GC-content was determined for each SNP using a window of 200bp (100bp flanking each SNP). Overlap with CpG Islands (CGI) was determined by using data from UCSC Genome Browser [67–69]. Overlap of the alleles with functionally-defined regions was mostly done with tracks provided by the ENCODE project [70]. This includes tracks of open chromatin (defined by the DNase I tracks) as well as 9 different modifications of histone H3 (K27me3, K4me1, K4me3, K4me2, K9ac, K27ac, K79me2, K36me3, K9me3). For all ENCODE data, an overlap was called when the respective track overlap was detected in any of the tissues and cell types deposited [70]. DMC1 ChIP-Seq tracks generated from human testis (marking meiotic recombination hotspots) were obtained from Pratto et al. [20]. Tracks of 5-methylcytosine (5mC) and 5-hydroxymethylcytosine (5hmC) generated from the embryonic stem cell line H1 were obtained from NIH Roadmap Epigenomics [1] and Szulwach et al. [21], respectively.

### Analysis of epigenetically defined sub-regions

111 reference epigenomes divided into 15 epigenetically defined sub-regions were obtained from the NIH Roadmap Epigenomics Consortium [1]. For each reference genome, the average NCO rate (1 - D’^2^_a,hap_) was computed separately for each sub-region using the LWK-based NCO rate each SNP had been assigned with. The same procedure was applied to define the state of chromatin (DNase I and 5mC), the presence of 5hmC marks, and histone marks (H3K27me3, H3K4me3, H3K36me3, and H3K9me3). Histone marks were defined using the respective tracks provided for each of the reference epigenomes. State of chromatin and 5hmC marks were calculated using the tracks defined for H1 stem cells [1, 21]. Definition of alleles located proximal or distal to recombination hotspots was based on the overlap with the testis-derived DMC1 tracks obtained from Pratto et al. (2014) [20].

### Statistical analysis and data visualization

Data processing and management was done using a combination of Biovia Pipeline Pilot and the R statistical language (version 3.3.1). Statistical analyses were done using the R statistical language (specifics of the statistical tests are described in the respective figure and table legends). Visualization of the data was done using both TIBCO Spotfire and R.

## Supplementary Information

**Additional file 1: Figure S1.** GC bias and frequency of archaic and ancestral haplotypes in the LWK cohort. (a) GC bias of NCO recombination. The transmission of A:T (red) or G:C (blue) alleles is shown for a subset of archaic SNPs heterozygous for A:T / G:C alleles (see methods section). Separate plots are shown for non-CpG and CpG SNPs; numbers represent the respective GC bias. (b) Relative frequency of archaic and ancestral haplotypes. The block diagram illustrates the absolute number of haplotypes of archaic linkage blocks that are representing perfectly preserved ancestral (green), perfectly preserved derived (blue) or mixed haplotypes (grey). The latter consist of both derived and ancestral alleles and are binned according to the relative fraction of derived alleles per haplotype. Horizontal lines represent the median. **Figure S2.** Impact of fitness on the apparent NCO recombination rate. Throughout the study D’ was used as proxy for the recombination rate. The parameter is mostly indicative on the number of haplotype variations present in a population, which could be influenced natural selection. The latter should be reflected in systematic changes in the allele frequencies of the respective SNP. In order to determine if fitness plays in fact a major role we therefore analysed the absolute frequency f_a_ of all derived alleles (grey bars) and the normalized frequency *Δ*f_a,hap_, representing the difference in frequency between the derived allele of singleton SNPs and their associated core haplotype (coloured bars) in reference to their predicted fitness contribution. (a) Fitness vs. function. Allele frequencies of the archaic SNP set are plotted against the scores of the function-related GWAVA- (left panels) and the fitness-related fitCons-database (right panel). Separate plots are shown for the absolute frequency f_a_ of all derived alleles (grey bars) and the normalized frequency *Δ*f_a,hap_. Blue bars represent retracting derived alleles (*Δ*f_a,hap_ < 0), green bars expanding derived alleles (*Δ*f_a,hap_ > 0); red bars comprise neutral alleles (*Δ*f_a,hap_ = 0), mostly representing the alleles of the core haplotypes. Each bar indicates the average score of the respective allele frequency bin; dashed lines indicate the average score of the entire dataset. While the absolute allele frequency of derived alleles (f_a_) did not yield any association, the correlation with Δf_a,hap_, revealed a positive association with GWAVA-scores, evident for both expanding (Δf_a,hap_ > 0) and retracting alleles (Δf_a,hap_ < 0). Importantly, fitCons scores showed no association with f_a_ or Δf_a,hap_. (b) Fitness inside and outside the HLA gene region. The correlation of fitCons scores with the normalized allele frequency *Δ*f_a,hap_ is shown for derived alleles located within or outside of HLA genes (MHC class I and class II). For HLA genes a clear correlation was evident for both positively and negatively selected alleles. Outside of the HLA region however only marginal effects were detected. (c) *Δ*f_a,hap_ histograms of coding alleles. The histograms display the relative frequency of coding SNPs binned according to their normalized allele frequency *Δ*f_a,hap_. A comparison is shown for derived alleles located within or outside of HLA genes, displaying the relative frequencies for all coding SNPs (left panel), synonymous coding SNPs (middle panel) and non-synonymous coding SNPs (right panel). A biased allelic expansion towards non-synonymous coding SNPs (indicative of natural selection) was evident inside, but not outside of this region,. As the MHC genes are the well-known exception of neutral evolution [71], our analyses are thus consistent with the concept of neutral evolution. According to this model, haplotypes are formed primarily by chance rather than by direct selection, implying that the number of allelic haplotype variants outside of the MHC is mostly influenced the recombination rate rather than selection. **Figure S3.** Dependency of NCO recombination rate on GC content, CpG islands and bivalent regions. (a) Local GC content. The average NCO-rate (1 – D’^2^_a,hap_) is correlated with the relative GC content of a 200bp window surrounding the SNP. Grey bars indicate the number of archaic SNPs in the respective GC content-bin subdivided into regions of closed chromatin (light grey), open chromatin (middle grey) or CpG islands (CGI; dark grey). (b) CpG islands (CGI). The average NCO-rate (1 - D’^2^_a,hap_) is plotted in reference of their distance to the boundary of CGI [67]. The plot covers alleles located inside (red) and outside (blue) of CGI; grey peak represents the GC content for the respective distance bin. (c) Bivalent regions. The average NCO-rate (1 - D’^2^_a,hap_) is shown for alleles located in bivalent regions characterized by overlapping tracks of both H3K4me3 and H3K27me3 (hatched bar), univalent regions characterized by overlapping tracks of either H3K27me3 (blue bar) or H3K4me3 (green bar) or regions without overlap to the tracks of any of the two marks (grey bar). Asterisks indicate significant differences between the indicated bars (*p* < 0.05; Mann-Whitney U test). **Figure S4.** NCO-rate and histone marks in epigenetic sub-regions. NCO rate and relative frequency histone marks are shown for 15 epigenetic sub-regions defined by the NIH Roadmap Epigenomics Consortium [1]. The calculations were carried out separately for each of 111 reference genomes using the entire set of 1.9 million archaic SNPs. (a) NCO recombination rate. The NCO recombination rate, expressed as average NCO-rate (1 - D’^2^_a,hap_), was calculated using the NCO-rate parameters defined for the LWK cohort [15, 16]. The calculation was carried out independently for each reference genomes by using the respective genome-specific region assignments. The boxplots represent the variation of average NCO-rate within these genomes; bars indicate the average NCO-rate of each sub-region. (b) Histone marks. The dot plots indicate the local enrichment of the histone marks H3K27me3, H3K4me3, H3K36me3 and H3K9me3. The dots represent the local enrichment of the marks in each of the reference epigenomes. The enrichment is expressed by the relative frequency of archaic alleles overlapping with the tracks (provided by the NIH Roadmap consortium [1]) in the respective sub-region. **Figure S5.** Enrichment of 5hmC in genomic sub-regions. The local 5hmC enrichment in various annotated regions of the human genome is shown. (a) Bivalent and DNase I assessable regions, CpG islands and DMC1 tracks. The bar charts indicate the 5hmC enrichment in bivalent regions (“bivalent”), open chromatin (“DNase I”), CpG islands (“CGI”) and meiotic recombination hotspots (“DMC1”). The location of bivalent regions (H3K27me3 & H3K4me3) and open chromatin (DNase I) was defined by ENCODE tracks; the location of CGI was defined by UCSC Genome Browser. Hotspot association was defined by the overlap with DMC1 tracks from human testis [20]. For each bar combination, the difference is significant (*p* < 0.05; Chi-square test). (b) Recombination rate and 5hmC enrichment of in GC-rich regions. The plots display the NCO-rate 1 – D’^2^_a,hap_ (red lines) and the relative enrichment of 5hmC marks (blue lines) in reference to the local GC-content (left panel) or the distance to CpG islands (right panel). (c) Frequency of 5hmC in gene regions. The NCO-rate of 1.9 million archaic SNPs was calculated for LWK; the 5hmC enrichment of the bin was determined by calculating the relative frequency of archaic alleles overlapping with published 5hmC tracks of H1 cells [21]; dashed lines represent the average values for the entire data set. Left panel: The local 5hmC enrichment is plotted in reference to the distance to the boundary of the closest annotated gene. Peaks are evident at 5′ or 3′ boundaries (indicated by dashed vertical lines). Genic regions are marked in red, intergenic regions in blue; dashed horizontal line represents the average overlap with 5hmC tracks of the entire set of archaic alleles. Right panel: The local 5hmC enrichment is shown for genic sub-regions (red: 5′ UTR, intron, splice site, exon and 3′ UTR); intergenic regions (blue) were binned according to the distance to the respective gene boundary (0-5kb, 5-50kb, <50kb). Horizontal dashed line represents the average 5hmC-overlap of the entire set of archaic alleles. For each bar combination, the difference is significant (*p* < 0.05; Chi-square test); the corresponding NCO recombination rate is shown in Fig. 2c,d.

**Additional file 2: Table S1.** Summary of data sources.

**Additional file 3: Table S2.** Details of the Mann-Whitney test statistics on the impact of epigenetic marks on the NCO recombination rate in genomic subregions (Fig. 3A). A description of all the columns in the table is provided in the worksheet "Description" of the ‘supplemental Table 2.xlsx’ file.

## Data Availability

The datasets analyzed during the current study are from published reports and are publicly available in the Additional file 2: Table S1 and are also listed below. ● The 1000 Genomes Project Consortium: An integrated map of genetic variation from 1,092 human genomes. Nature 2012, 491(7422):56-65. https://www.internationalgenome.org/ ● Roadmap Epigenomics Consortium, Kundaje A, Meuleman W, Ernst J, Bilenky M, Yen A, Heravi-Moussavi A, Kheradpour P, Zhang Z, Wang J et al: Integrative analysis of 111 reference human epigenomes. Nature 2015, 518(7539):317-330. https://egg2.wustl.edu/roadmap/web_portal/ ● Ritchie GRS, Dunham I, Zeggini E, Flicek P: Functional annotation of non-coding sequence variants. Nature methods 2014, 11(3):294-296. https://www.sanger.ac.uk/sanger/StatGen_Gwava ● Gulko B, Hubisz MJ, Gronau I, Siepel A: A method for calculating probabilities of fitness consequences for point mutations across the human genome. Nature genetics 2015, 47(3):276-283. http://compgen.cshl.edu/fitCons/ ● Sloan CA, Chan ET, Davidson JM, Malladi VS, Strattan JS, Hitz BC, Gabdank I, Narayanan AK, Ho M, Lee BT et al: ENCODE data at the ENCODE portal. Nucleic acids research 2016, 44(Database issue):D726-D732. https://www.encodeproject.org/ and https://genome.ucsc.edu/encode/ ● Karolchik D, Barber GP, Casper J, Clawson H, Cline MS, Diekhans M, Dreszer TR, Fujita PA, Guruvadoo L, Haeussler M et al: The UCSC Genome Browser database: 2014 update. Nucleic acids research 2014, 42(Database issue):D764-770. https://genome.ucsc.edu/ ● Pratto F, Brick K, Khil P, Smagulova F, Petukhova GV, Camerini-Otero RD: Recombination initiation maps of individual human genomes. Science 2014, 346(6211). 10.1126/science.1256442 ● Szulwach KE, Li X, Li Y, Song C-X, Han JW, Kim S, Namburi S, Hermetz K, Kim JJ, Rudd MK et al: Integrating 5-Hydroxymethylcytosine into the Epigenomic Landscape of Human Embryonic Stem Cells. PLOS Genetics 2011, 7(6):e1002154. 10.1371/journal.pgen.1002154 The complete dataset used in this study has been deposited as a tab-delimited text file together with an Excel metadata file describing all the fields within the tab-delimited text file at: ● Lee, B. Analysis of archaic human haplotypes suggests that 5hmC acts as an epigenetic guide for NCO recombination. 2021. 10.7910/DVN/WA7IUK

## Abbreviations

5hmC: 5-Hydroxymethylcytosine
5mC: 5-Methylcytosine
BivFlank: Bivalent flanking region
bp: Base-pairs
CGI: CpG Islands
ChIP-seq: Chromatin Immunoprecipitation and Sequencing
CO: Crossover
DDR: DNA damage repair
*Denisovan h.*: *Denisovan hominid*
DMC1: Meiotic recombination protein, RecA homolog
ENCODE: Encyclopedia of DNA elements
EnhBiv: Bivalent enhancer
f_a_: Absolute frequency of derived alleles
FitCons: Fitness consequence
GWAVA: Genome-wide annotation of variants
*H. sapiens*: *Homo sapiens*
hap: Core haplotype
HLA: Human Leukocyte Antigen
kb: Kilo-basepairs
LD: Linkage disequilibrium
LWK: Luhya from Webuye, Kenya
MHC: Major histocompatibility complex
NCO: Non-crossover
PRC2: Polycomb repressive complex 2
ReprPC: Polycomb repressed regions
ReprPCWk: Weak polycomb repressed regions
SNP: Single nucleotide polymorphism
TEI: Transgenerational epigenetic inheritance
TSS: Transcriptional start site
TssBiv: Bivalent transcriptional start site
Tx: Transcribed region
TxFlank: Flanking a transcribed region
TxWk: Weakly transcribed region
UTR: Untranslated region
ZNF: Zinc finger
