# Supplementary material for: Analysis of archaic human haplotypes suggests that 5hmC acts as an epigenetic guide for NCO recombination
Source: BMC Biol. 2022 Aug 4;20:173. doi: 10.1186/s12915-022-01353-9 (PMC9354366; doi:10.1186/s12915-022-01353-9)
Supplement: Supplementary file 1 — Additional file 1: Figure S1. GC bias and frequency of archaic and ancestral haplotypes in the LWK cohort. (a) GC bias of NCO recombination. The transmission of A:T (red) or G:C (blue) alleles is shown for a subset of archaic SNPs heterozygous for A:T / G:C alleles (see methods section). Separate plots are shown for non-CpG and CpG SNPs; numbers represent the respective GC bias. (b) Relative frequency of archaic and ancestral haplotypes. The block diagram illustrates the absolute number of haplotypes of archaic linkage blocks that are representing perfectly preserved ancestral (green), perfectly preserved derived (blue) or mixed haplotypes (grey). The latter consist of both derived and ancestral alleles and are binned according to the relative fraction of derived alleles per haplotype. Horizontal lines represent the median. Figure S2. Impact of fitness on the apparent NCO recombination rate. Throughout the study D’ was used as proxy for the recombination rate. The parameter is mostly indicative on the number of haplotype variations present in a population, which could be influenced natural selection. The latter should be reflected in systematic changes in the allele frequencies of the respective SNP. In order to determine if fitness plays in fact a major role we therefore analysed the absolute frequency fa of all derived alleles (grey bars) and the normalized frequency Δfa,hap, representing the difference in frequency between the derived allele of singleton SNPs and their associated core haplotype (coloured bars) in reference to their predicted fitness contribution. (a) Fitness vs. function. Allele frequencies of the archaic SNP set are plotted against the scores of the function-related GWAVA- (left panels) and the fitness-related fitCons-database (right panel). Separate plots are shown for the absolute frequency fa of all derived alleles (grey bars) and the normalized frequency Δfa,hap. Blue bars represent retracting derived alleles (Δfa,hap < 0), green bars expand [file 12915_2022_1353_MOESM1_ESM.pptx]

## Slide 1
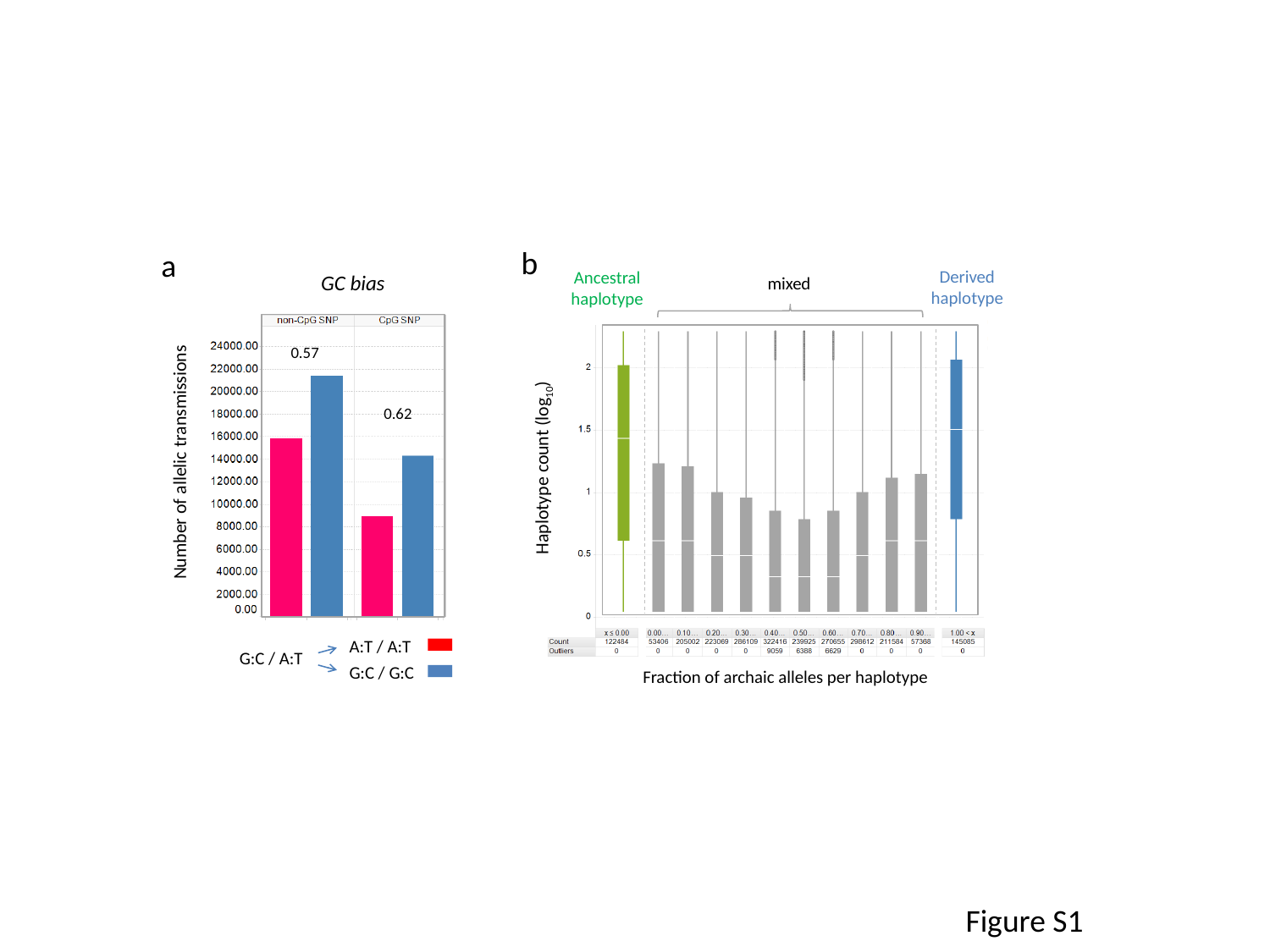

b
Derived
haplotype
Ancestral
haplotype
mixed
Haplotype count (log10)
Fraction of archaic alleles per haplotype
a
GC bias
0.57
0.62
Number of allelic transmissions
A:T / A:T
G:C / G:C
G:C / A:T
Figure S1

## Slide 2
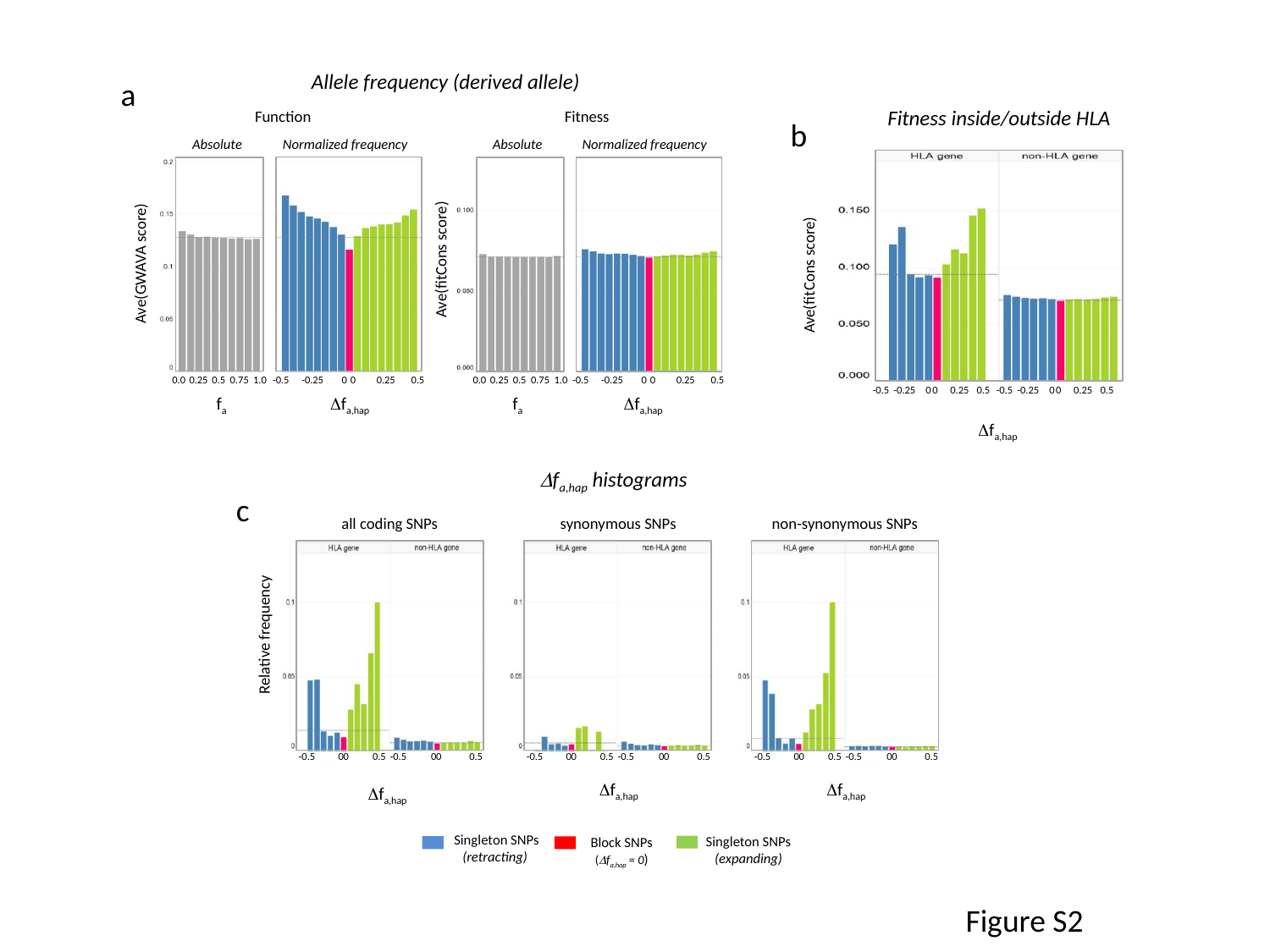

Allele frequency (derived allele)
a
Function
Fitness
Absolute
fa
Ave(fitCons score)
0.0
0.25
0.5
0.75
1.0
Normalized frequency
Dfa,hap
-0.5
-0.25
0
0
0.25
0.5
Absolute
fa
Ave(GWAVA score)
0.0
0.25
0.5
0.75
1.0
Normalized frequency
Dfa,hap
-0.5
-0.25
0
0
0.25
0.5
Fitness inside/outside HLA
b
Ave(fitCons score)
-0.5
-0.25
0
0
0.25
0.5
-0.5
-0.25
0
0
0.25
0.5
Dfa,hap
Dfa,hap histograms
c
all coding SNPs
synonymous SNPs
non-synonymous SNPs
Relative frequency
Dfa,hap
Dfa,hap
Dfa,hap
-0.5
0
0
0.5
-0.5
0
0
0.5
-0.5
0
0
0.5
-0.5
0
0
0.5
-0.5
0
0
0.5
-0.5
0
0
0.5
Singleton SNPs
(retracting)
Singleton SNPs
(expanding)
Block SNPs
(Dfa,hap = 0)
Figure S2

## Slide 3
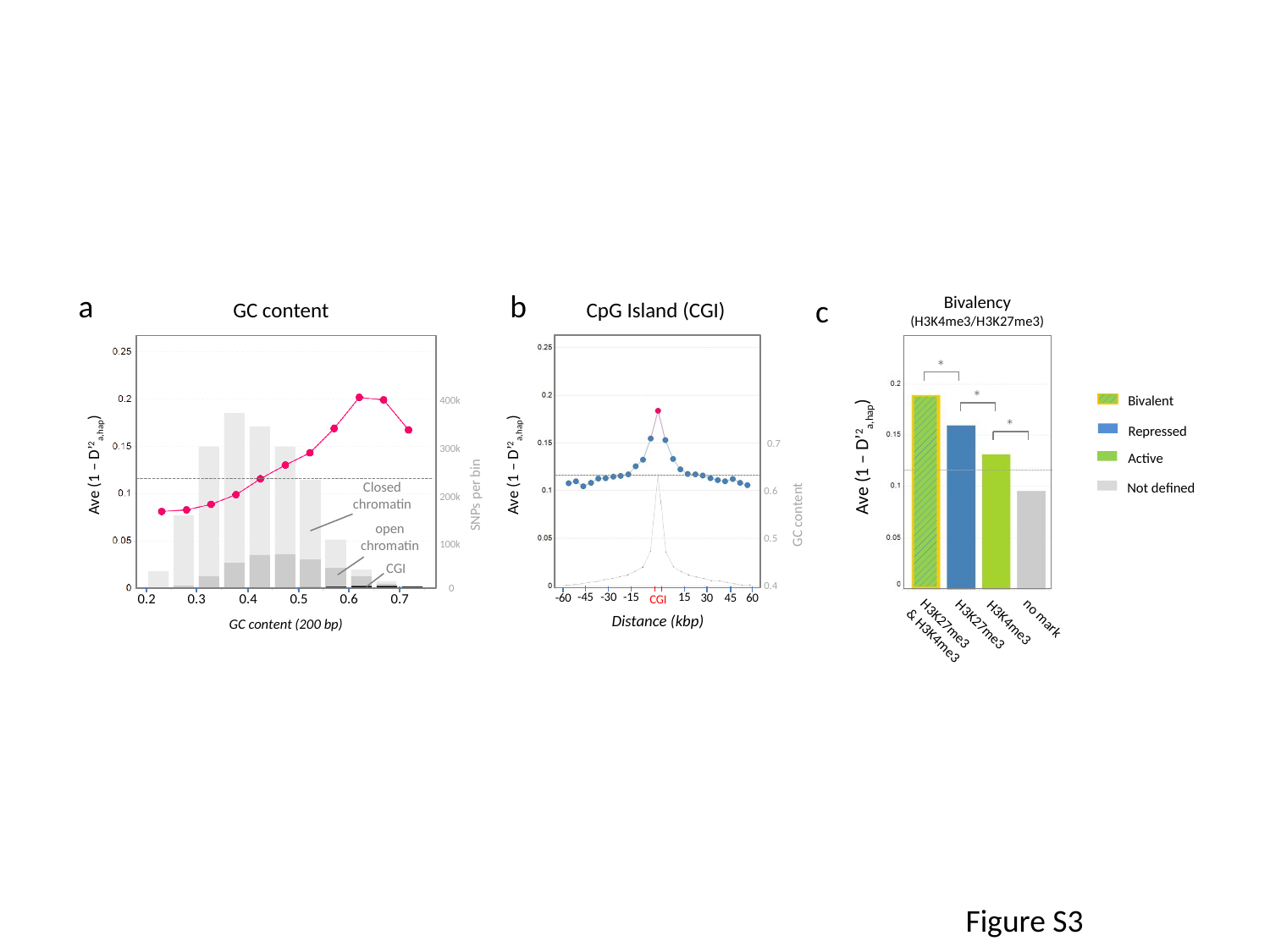

a
b
c
Bivalency
(H3K4me3/H3K27me3)
Ave (1 – D’2a,hap)
no mark
H3K27me3
& H3K4me3
H3K4me3
H3K27me3
Bivalent
Repressed
Active
Not defined
*
*
*
CpG Island (CGI)
GC content
400k
300k
SNPs per bin
200k
100k
0.7
 Ave (1 – D’2a,hap)
 Ave (1 – D’2a,hap)
Closed chromatin
0.6
GC content
open chromatin
0.5
CGI
0.4
0
-45
-30
-15
15
0.7
30
45
60
-60
0.2
0.3
0.4
0.5
0.6
CGI
Distance (kbp)
GC content (200 bp)
Figure S3

## Slide 4
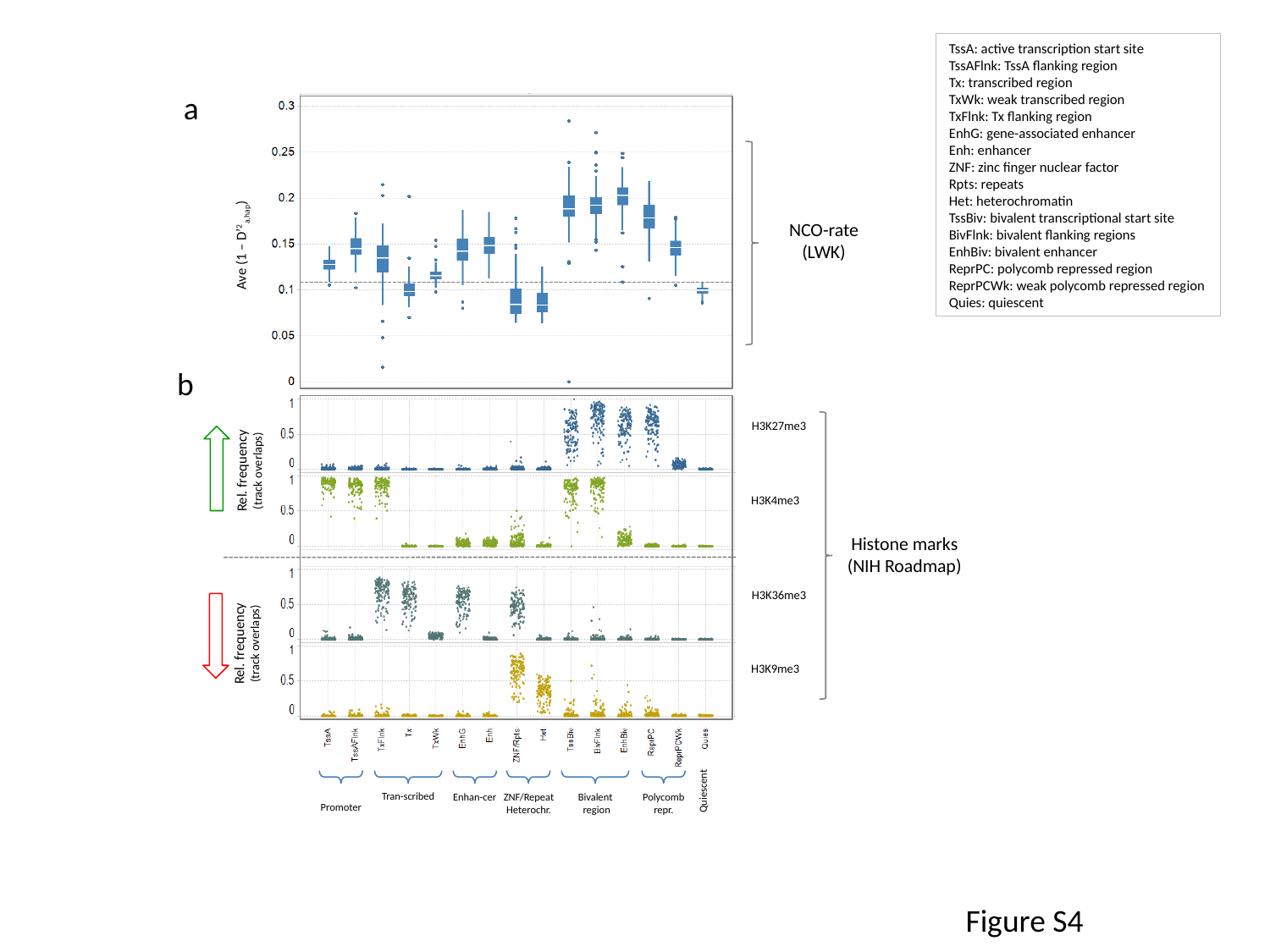

TssA: active transcription start site
TssAFlnk: TssA flanking region
Tx: transcribed region
TxWk: weak transcribed region
TxFlnk: Tx flanking region
EnhG: gene-associated enhancer
Enh: enhancer
ZNF: zinc finger nuclear factor
Rpts: repeats
Het: heterochromatin
TssBiv: bivalent transcriptional start site
BivFlnk: bivalent flanking regions
EnhBiv: bivalent enhancer
ReprPC: polycomb repressed region
ReprPCWk: weak polycomb repressed region
Quies: quiescent
a
NCO-rate (LWK)
Ave (1 – D’2a,hap)
b
H3K27me3
Rel. frequency
(track overlaps)
H3K4me3
Histone marks
(NIH Roadmap)
H3K36me3
Rel. frequency
(track overlaps)
H3K9me3
Quiescent
Tran-scribed
ZNF/Repeat
Heterochr.
Bivalent
 region
Polycomb
repr.
Enhan-cer
Promoter
Figure S4

## Slide 5
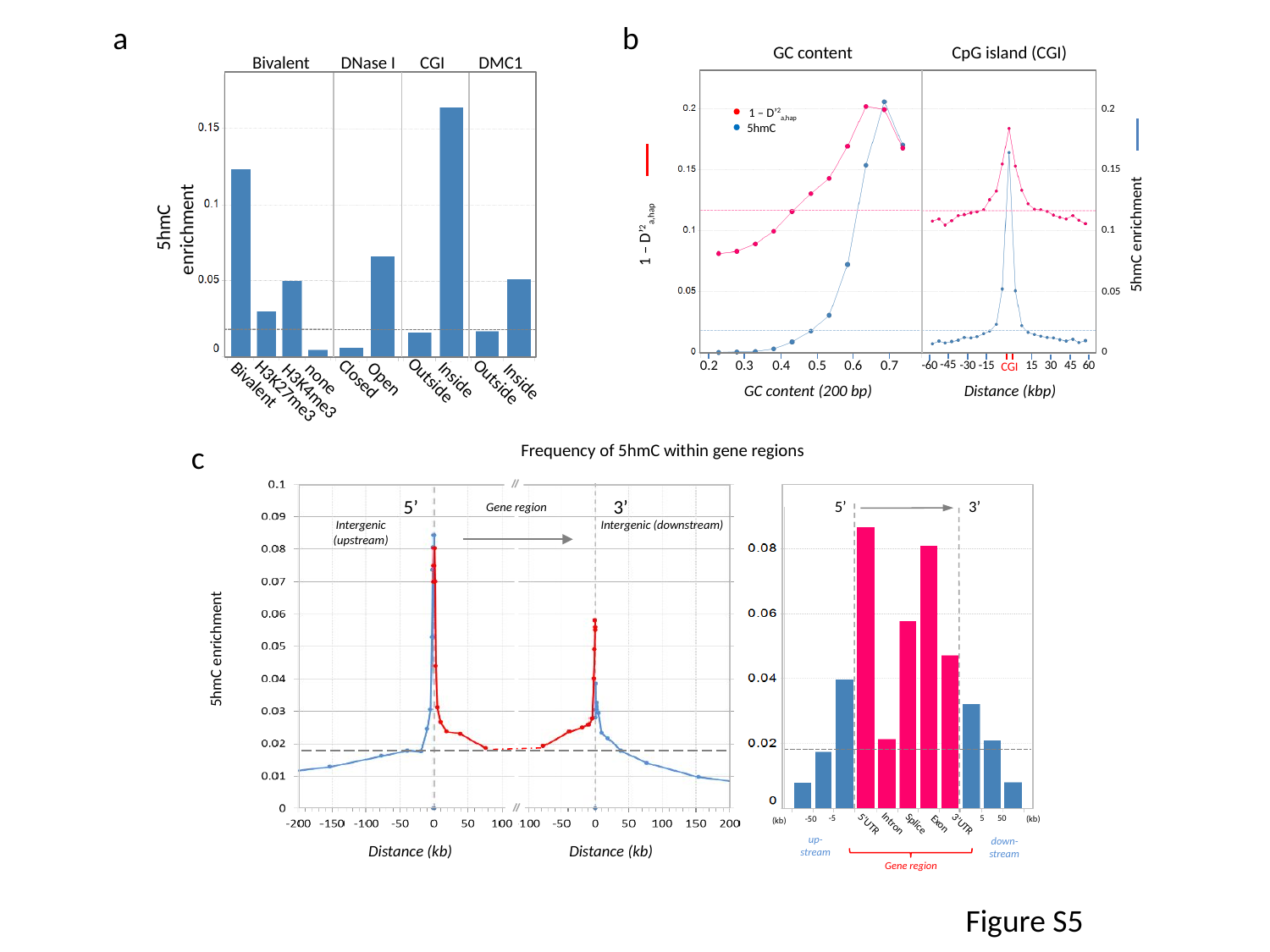

a
b
GC content
CpG island (CGI)
0.2
0.15
0.1
0.05
0
1 – D’2a,hap
5hmC
5hmC enrichment
1 – D’2a,hap
0.7
0.2
0.3
0.4
0.5
0.6
GC content (200 bp)
-45
-30
-15
15
30
45
60
-60
CGI
Distance (kbp)
CGI
DMC1
Bivalent
DNase I
 5hmC enrichment
none
Bivalent
H3K4me3
H3K27me3
Inside
Outside
Inside
Outside
Open
Closed
c
5’
3’
Gene region
Intergenic (upstream)
Intergenic (downstream)
Distance (kb)
Distance (kb)
 5hmC enrichment
Frequency of 5hmC within gene regions
5’
3’
Splice
Exon
Intron
5’UTR
3’UTR
5
50
-5
-50
up-
stream
down-
stream
Gene region
(kb)
(kb)
Figure S5
